# “*It’s a waste of time coming here, better go to private clinics with wider options for treatment”*: patient’s perception on dental services provided in Fiji

**DOI:** 10.1186/s12913-022-08543-9

**Published:** 2022-09-10

**Authors:** Richard D. Nair, Masoud Mohammadnezhad

**Affiliations:** 1 School of Public Health and Primary Care, Fiji National University, Suva, Fiji; 2 Faculty of Health Studies, School of Nursing and Healthcare Leadership, University of Bradford, West Yorkshire BD7 1DP Bradford, UK

**Keywords:** Patients perception, Dental clinic, Quality of services, Fiji

## Abstract

**Background:**

Oral health can influence the quality of an individual’s life. Patient’s perception of the service plays a vital role in understanding the reasons as to why a patient may be satisfied or dissatisfied with the service that they accessed at a dental setting. As no studies have been done in Fiji until now, this study aimed to explore the perceptions of patients on services provided by the largest dental clinic in the Central/Eastern division in Fiji.

**Method:**

A qualitative study was used to collect data from patients who visited the Colonial War Memorial Hospital (CWMH) dental clinic in Suva Fiji from 5^th^ August to 31^st^ October, 2020. All patients above the age of 18 of both genders and from any ethnicity who visited the CWMH dental clinic during the period of data collection were included the study. A total of 25 participants were interviewed for this study using the in-depth interview method till data saturation occurred. A semi-structured open-ended questionnaire was used to collect data using face-to-face in-depth interviews. The data were transcribed and analyzed using manual thematic analysis process to gather the themes and sub-themes for the results.

**Results:**

A total of 25 patients were interviewed, with a majority (*n* = 14) being men and 15 were of I-Taukei background. Five themes emerging from data analysis include: Waiting time before treatment, Cost of Treatment, Accessibility of services, Privacy and confidentiality and Range of treatment options. The patients had an expectation to get the best treatment but face many hurdles while trying to get the treatment that they expect. The shortfalls on the part of this dental clinic caused an onset of negative perception among its users.

**Conclusion:**

This study shows an overall dissatisfaction with regards to services delivery among the patients who use the CWMH dental clinic for dental care purposes. The decision makers need to look into the genuine concerns that have been raised by patients in order to create improvements in services delivery and create an array of satisfaction for its patients.

## Background

Oral health can influence the quality of an individual’s life [1]. According to the World Health Organization (WHO) oral health has been identified as a key indicator which influences overall health, well-being, and Quality of Life (QoL). In terms of diseases, oral health conditions encompass dental caries, periodontal disease, tooth loss, oral cancer, oral manifestations of human immunodeficiency virus infection, oro-dental trauma, noma and birth defects such as cleft lip and palate [2].

WHO additionally mentions that in a study conducted in 2017 on the global burden of disease, oral diseases affect 3.5 billion people worldwide, with untreated dental caries being among the most prevalent Non Communicable Diseases (NCDs) [2]. Furthermore, it was established by the International Agency for Research on Cancer (IARC) that oral cancer was in the top three categories of all cancers in most Asian Pacific Countries in 2018 [3].

Maintenance of good oral health therefore becomes a priority to stay healthy, but in extremes when the oral health of a person is compromised, as mentioned in the charter of the WHO, it is the duty of a dental practitioner to restore the oral health to the satisfaction of the patient [2]. In gauging the satisfaction, the patient’s perception of the service plays a vital role in understanding the reasons as to why a patient may be satisfied or dissatisfied with the service that they accessed at a dental setting [4].

Akbar et al. [5] in their study mention that patient’s perception is generally accounted as patients’ experience about the service given to them. This perception has the probability to affect satisfaction and influence the patient's cooperation level and enable the dentist in arranging future schedules for the well-being of the patients. This was further elaborated in a study conducted by Karydis A et al. [6] which found that patients who had a bad experience with the dental practitioner defaulted attending a follow up clinic which was important for them to ensure good oral health.

Expectations and perceptions are the main things that determine patient satisfaction that forms the basis of evaluating the quality of services provided. To gain satisfaction with the service, the perceptions felt by the patient must be at least equal to or more than their expectations [5]. Furthermore, a study done in Iran by Bahadori et al. [7] on the barriers affecting access to dental services indicated that there were five determinants that affected patient access and perception which included cost, inconvenience, fear, organization, and patient-dentist relationship, among which the cost and patient-dentist relationship were identified as the first and last priorities with the coordinates (1.4 and 1.4) and (1.25 and -0.65); respectively. This fear factor drove patients away from accessing services, which caused serious oral health issues.

Furthermore, Nair et al. [8] found that patient’s perceptions were primarily based on their experience with dentists and the way services were delivered in a clinical setting. The authors also concluded that negative perceptions were linked to the cost of dental services, poor accessibility, dental anxiety, and fear of pain endured during treatment.

Patients visit a dental clinic particularly a public facility to access dentistry services at a reasonable cost and without compromised care and have a certain perception of the way in which they are treated at the clinic, and most of the time this perception tends to take a negative approach by the way they are treated by the dentists. As no studies have been done in Fiji until now, this study aimed to explore the perceptions of patients on services provided by Colonial War Memorial Hospital (CWMH) dental clinic the largest dental clinic in the Central/Eastern division in Fiji.

## Methodology

### Study design and setting

This study used a qualitative design to explore the perceptions of patients on the services provided by the CWMH dental clinic, Suva Fiji from 5^th^ August to 31^st^ October, 2020. This clinic was selected for this study because it is attached to the bigger CWMH hospital and is the largest dental clinic in the Central/Eastern division located centrally near the city of Suva and also caters for referral cases from other satellite dental clinics located within the division. The clinic has 12 dental chairs and a waiting area where patients are given a number and they wait for their turn to be seen by the dental staff. This dental clinic is run by the Ministry of Health and Medical Services and can be classified as a government run clinic where patients can access basic services dental services at a lower cost in comparison to private dental clinics since the government has subsidized the cost to allow affordable dental care for all its citizens.

### Study sample

The population of this study included all the patients who attended the dental clinic at CWMH dental clinic. All patients above the age of 18 of both genders and from any ethnicity who visited the CWMH dental clinic during the period of data collection were included the study while the exclusion criteria encompassed any patient who was unwilling to participate in the interview due to personal reasons or because of health issues which came about after receiving treatment as well as those patients who were visiting the clinic for the first time as they would not have enough experience of the dental clinic. A total of 25 participants were interviewed for this study using the in-depth interview method till data saturation occurred. According to Fusch and Ness [9] failure to hit data saturation has a negative effect on the study quality and material validity. When there is enough information to reproduce the analysis, the opportunity to collect additional new information has been achieved, and more coding is no longer possible, data saturation has occurred. In this study after the 25th participant had been interviewed it was determined that no new information was being gathered, thus data saturation was achieved and the interview was concluded.

### Data collection tool

The study employed the in-depth interview method whereby face-to-face interviews on a one-to-one basis was done using semi structured questionnaire which was designed for this study by using relevant literatures and research studies [3, 4, 7]. The in-depth interviews among the participants consisted of eight open ended semi- structured questions. The semi structured questionnaires also had a section for demographic information to capture the details of the participants being interviewed. The open-ended interviews allow participants to discuss their opinions, views, and experiences fully in detail [10]. This enables the researcher to explore the lived experiences of participants without contaminating the data.

### Study procedure

Patients who had been seen by the dentists at the clinic were approached politely by the researcher at the CWMH dental clinic waiting area and were asked if they were willing to participate in the study. Once they agreed to take part in the study and prior to the start of the interview, the patients were informed by the researcher that the conversation will be recorded using a voice recorder. An information sheet was provided to each participant for the interview for them to read and keep for future reference if the need arises. The contents of the information sheet was also explained to the participants in detail if someone was unable to read, and the information sheet was also prepared in the Hindi and I-taukei language should some participants prefer reading this in their own language but this was not the case as all the participants were English speaking and preferred English as the form of communication for this interview. A written informed consent form was also filled by the participants before the start of the interview giving the researcher permission to conduct the interview. Demographic information such as age, gender, and ethnicity was captured first before the start of the interview.

The in-depth interviews for the patients lasted between 20–30 min depending on the participant’s responses which determined the direction and length of the interview for this research. The interviews for patients was carried out by the main researcher of this study over a period of 3 weeks (2 interviews a day), which allowed the researcher to reflect and make adjustments to the interview questions for example more questions were added or some questions were removed after the first round of interviews based on their relevance. The interviews with the participants, was conducted at the dental clinic in a quiet room away from distractions.

### Data management and analysis

The interviews were transcribed by the researcher of this study using verbatim transcription. A verbatim transcript captures each and every word from an audio file in print, exactly as it was spoken at the time [11]. The notes taken during the interview by the researcher was also compared with the transcriptions to ensure that similar information was captured. All the data collected in terms of voice recordings was transcribed in a word file by the researcher to ensure that quality is maintained.

Analysis of the transcripts was done by the researcher using thematic analysis. Thematic analysis is a method used for analyzing qualitative data and is applied to set of texts such as interview transcripts. In thematic analysis the researcher carefully examines the data to identify common themes topics, ideas and patterns of meaning that come up repeatedly [12]. The participant’s responses were read and re-read closely by the main researcher to divide into key words or phrases into their similar meanings and create codes. The transcribed results were coded and sorted into themes and sub-themes based on the similar issues which formed the result of the study. For this research the 6 steps of thematic analysis as described by Braun and Clarke, 2006 in their journal paper “Using thematic analysis in psychology” was used.

### Study rigor

Rigor or trustworthiness of a study is defined as the degree of confidence in data, interpretation and approaches used to ensure the quality of study [13]. Cypress [14] defines rigor as establishing trust in the findings of a research study. Trustworthiness was incorporated in this study by using Lincoln and Guba [15] strategies of credibility, transferability, dependability, and conformability.

In terms of the credibility of this study, a comprehensive background of the research had been provided explaining the importance of this study and the necessary approvals were obtained from the respective agencies have been included for reference. Transferability was established in this study by providing background information to create the context of the study and providing a thorough explanation of the phenomenon in question so that related research can be compared. Dependability was addressed in this research by providing an in-depth methodological description to allow this study to be repeated. Conformability was assured as there was no investigator bias as the main researcher is not from the dentistry field and is in no way involved in any dental related activities concerning the profession, thus there is no investigator bias or any conflict of interest, plus an in-depth methodological description was provided to allow integrity of research findings to be scrutinized.

## Results

A total of 25 patients, took time to participate in this study whereby 14 were males and 11 were females. The participants were between the age group of 20–60 years. With respect to ethnicity 15 were of I-Taukei background while 10 were Fijians of Indian descent. The Marital status breakdown included 5 single participants and 20 participants were married (Table 1).

**Table 1:** Demographic characteristics of participants (*n* = 25)

Personal information	Frequency	Percentage (%)
**Gender**		
Male	14	56
Female	11	44
**Age (years)**		
20–30	3	12
31–40	12	48
41–60	10	40
**Ethnicity**		
I-Taukei	15	60
Fijians of Indian Origin	10	40
**Marital Status**		
Single	5	20
Married	20	80

### Theme identified

The thematic analysis of the interview found the 5 themes emerging from the main theme of perception of dental services. The Five main themes identified for perception of dental services include: Waiting time before treatment, Cost of Treatment, Accessibility of services, Privacy and confidentiality and Range of treatment options (Table 2).

**Table 2:** Themes and codes identified

Theme	Open codes	Quotation examples
Waiting time before treatment	Long waiting time Staff on sick leave Need for more staff Option of private dentist Dentist’s relatives seen first School children should not be made to wait No Compassion	*“I came at 7am before they even opened the clinic…”* *“I was informed that most of the dental staff are on sick leave…”* *“I think this clinic needs more dental staff…”* *“I would prefer to see a private dentist even if it….”* *“The dentist’s relatives are seen first…”* *“School children should be given priority…”* *“No value for human pain here…”*
Cost of treatment	Tax payers Covid-19 Inferior treatment Government blamed Reasonable cost	*“We are tax payers so in a way we have already…”* *“Because of covid I feel it’s going to get tougher…”* *“They charge us fees but the filling they do comes out…”* *“May be the government could consider poor people like us…”* *“I don’t think the charges are that expensive…”*
Accessibility of services	Disabled and elderly access No parking space for disabled patients Grumpy staff Dental chair not working	*“For me coming up to CWM dental clinic is a challenge…”* *“It was an issue finding parking space…”* *“Look at the dentists we have they are so grumpy…”* *“I asked the dentist to lower the dental chair so that I could sit properly…”*
Privacy and confidentiality	Treatment room exposed Patient’s folders	*“The treatment room is not a room it is sort of open…”* *“The reception staff they carrying the patient folders as if…”*
Range of treatment options	Limited options Covid-19 Availability of dental materials Tooth loss Having qualified dentists and less treatment options	*“Some of the services are not available here…”* *“They said because of covid they only…”* *“Treatment will depend on the availability of materials…”* *“Today I came to get my tooth pulled out because 5 months ago…”* *“What’s the use of having so many qualified dentists when the clinic…”*

#### Theme 1: Waiting time before treatment

All the patients described the waiting time before seeing a dentist and receiving treatment as long and dissatisfying. The late start in the working time was considered as the reason for prolonged waiting time.

*“I was referred to the clinic from Nausori one day before, I came at 7am before they even opened the clinic, and I was seen by the dentist at 10am. I don’t know what took them so long… this is very frustrating as I have to go back to work and my pay will be cut for 2 hours”.* (P4, a 33 years old Indian male).

Staff taking sick leave on Monday’s was also a reason identified by participants that contributed to the waiting time.

*“The waiting time is bad at this clinic as I had to wait for 3 hours before I could be seen by a dentist. When I asked the front desk person the reason for the delay, I was informed that most of the dental staff are on sick leave. Monday fever I guess”.* (P13, a 37 years old I-Taukei, male)

Another participant highlighted the need for more dental staff and the slow work process of the current staff as something that increases waiting time at the clinic.

*“I think this clinic needs more dental staff as with the current staff they are very slow, I had to wait for an hour before being seen by the dentist*.” (P24, a 44 years old Indian, female).

The option of going to a private dentist was also mentioned by a participant to avoid the long waiting time.

*“The waiting time is so bad that next time I would prefer to see a private dentist even if it costs me a lot. I can’t waste my time waiting to receive dental treatment at this clinic.”* (P6, a 30 years old I-Taukei, male)

Likewise, a participant raised the issue that dentist’s relatives are seen first which again prolongs the waiting time of patients waiting in line for their turn.

*“Waiting time becomes longer for us because sometimes the dentists relatives are seen first, I saw that happen today the guy just walked in the waiting area and out came the dentist to take him inside. Who you know system is being practiced here.”* (P19, a 27 years old Indian, male)

P19’s comment was validated by, P7, a 44 years old I-Taukei, Female who stated:

*“I don’t have an issue with the waiting time as my relative is dentist here and I get seen fast.”*

It was also highlighted by some participants that children and school children should not be made to wait as they have to get back to school after receiving treatment.

*“School children should be given priority as they need to get treatment fast and get back to school, it was very frustrating having to wait for about an hour before my son could be seen by the dentist.”* (P20, a 38 years old I-Taukei, female)

Similarly, another participant stated.

*“My 5 year old son had a very bad tooth ache and was crying, I asked the receptionist if my son could be seen immediately as it was an emergency…. I was told to take a number and wait, I waited for 2 hours with my son who was in pain and the whole time crying. No compassion.”* (P9, a 25 years old I-Taukei, female)

Participants also stated that there was no compassion as dentists don’t have a sense of responsibility towards patients and are lazy, especially in government clinics.

*“We can’t expect much from the government dental clinic because it is government, never mind the dentist study in overseas but when working in Fiji all the same. Lazy and no sense of responsibility that’s why we poor patients have to suffer.”* (P18, a 35 years old I-Taukei, male)

*“No value for human pain here…. take a number, sit and wait for your turn never mind you are about to die of that tooth pain. It can take about an hour before one is seen by the dentist”* (P12, a 31 years old I-Taukei, female)

#### Theme 2: Cost of treatment

The CWMH dental clinic is a government funded facility, but since the dental materials procurement becomes expensive, cost of certain services provided needs to be paid by the patients at a very subsidized rate.

Majority of the patients on the other hand feel that the cost of service is a bit too much and the idea of paying for a government service does not go down well from the perception of the participants as many are not earning enough to afford this service as mentioned by P2, a 45 years old Indian, Female:

*“I do not like the idea of paying for this service. This is part of the CWM hospital we don’t pay to see the doctor than why do we have to pay to see the dentist? This is just bad ripping poor people off.”*

Likewise,

*“Why do we have to pay for something that we have already paid for? We are tax payers so in a way we have already paid for the service even before using it. I think the government is broke that’s why charging us so much fees*.” (P23, a 55 years old Indian, male)

The recent Covid-19 pandemic which caused job loses, was also identified by participants as a reason why they find the cost of treatment expensive.

*“ I paid my dental fees but feel that it is a bit too much especially for us who don’t work and now because of covid I feel it’s going to get tougher to seek dental treatment with these types of fees in place.”* (P14, a 35 years old Indian, female)

The participants also identified the issue of inferior treatment being given to them and when they return they are asked to pay again for that service.

*“They charge us fees but the filling they do comes out in a few months when I returned as it is counted as return job they said I will have to pay again…… I don’t know if I can take this issue to consumer council or not but it’s very bad.”* (P16, a 33 years old Indian, male)

Some participants blamed the government for not caring for the poor elderly patients who have to borrow money to pay for dentures with the added burden of visiting the clinic many times before they get their dentures.

“*I needed dentures (false teeth) last year but could not pay for the cost. Iam old and retired civil servant I don’t have money in my pocket every day. I had to borrow money to so that I could get my dentures done, may be the government could consider poor people like us*.” (P1, a 60 years old I-Taukei, female)

While, (P3, a 58 years old Indian, male).

“*I am a Social Welfare recipient, now I have to travel all the way to Nakasi to get my dentures made, because the CWM denture making department closed, and I have to go to Nakasi plenty times before I get my Dentures…it’s not cheap you now this running around*”

Some participants on the contrary thought that the charges at CWMH dental clinic was reasonable in comparison to the private dental clinics as, P21, a 39 years old Indian, Male mentioned:

*“I don’t think the charges are that expensive. It’s affordable in comparison to the private dental clinics. They really rip us off.”*

#### Theme 3: Accessibility of services

Majority of the participants interviewed did not identify any issues with the accessibility of services apart from a few who had issues with disabled and elderly person’s access like:

*“For me coming up to CWM dental clinic is a challenge as I can’t walk properly plus I get off the bus and have to climb the stairs to get here. I think the clinic needs to be situated at a place which is disabled friendly.”* (P24, a 44 year old Indian, female)

Similarly,

*“My daughter is disabled as she can’t walk. The last time I brought her to this clinic it was an issue finding parking space so that I could get her off and then use her wheel chair to cart her to the clinic. I think the government needs to make this clinic disabled friendly and provide specified parking spots so that we don’t face any issues when getting our disabled family members to the clinic.”* (P15, a 36 years old Indian, male)

A participant mentioned that staff are grumpy with normal patients and wondered how the disabled people may be treated considering they are not able bodied.

*“For disabled people accessibility would be an issue. First in getting to this clinic and second in receiving treatment, look at the dentists we have they are so grumpy with us normal people imagine what they would do to a disabled person. I think that is one of the reasons why a disabled person does not seek dental treatment at government dental clinic.”* (P12, a 31 years old I-Taukei, female)

Another participant mentioned the dental chairs used by the dentists in this clinic that are not disabled friendly as it does not incline up and down.

*“This clinic has accessibility issues when it comes to disabled people. Just go inside and see the chairs that they work on is so bad it does not go up or down so how would a disabled person climb onto a chair that is high. Better to go to private clinics if one is disabled and needs dental care.”* (P2, a 45 years old Indian, female)

This was experienced by P5, a 60 years old I-Taukei, male who stated:

*“I am 60 years old and sometimes need dental care. Coming to this clinic is not easy due to its location and then the equipment’s that the dentists use. I asked the dentist to lower the dental chair so that I could sit properly but he said it does not go down because it is bad and not yet fixed. I had to sort of jump up as I am short and old and the unfortunate part is that the dentist just stood there watching did not even consider to help me…….. I would rather not come here again and embarrass myself never mind my teeth falls off that’s ok.”*

#### Theme 4: Privacy and confidentiality

Many of the participants interviewed did not identify any issues with the privacy and confidentiality. On the contrary there were a few participants who raised some crucial issues when it came to their privacy and confidentiality with regards to the treatment room and handing of patient folder the at this dental clinic like P12, a 31 years old I-Taukei, Female mentioned:

*“The treatment room is not a room it is sort of open from the bottom and the top so what the dentist is saying in the other room to his patient can be heard by the patient in the neighboring room. I think there needs to be closed rooms to maintain privacy.”*

Similarly, P16, a 33 years old Indian, Male stated:

*“They don’t even close the door, while the dentist was working on my tooth another dentist worked in to have a chat with the dentist while she was working on my tooth and all this was happening as I lay there with my mouth wide open. I felt embarrassed where is the privacy and confidentiality part which they are supposed to maintain.”*

In regards to the folder privacy issue at the clinic P11, a 36 years old I-Taukei, Male indicated:

*“Is there anything private and confidential in a government facility? Look the reception staff they carrying the patient folders as if it’s nothing. What if the folders contain vital information about a patient who has HIV/AIDS? The way it is being handled all information can leak out. This is the reality of a government hospital”*

#### Theme 5: Range of treatment options

Patients expressed concerns with regards to the range of treatment options provided and available at the CWMH dental clinic, with some stating that options are very limited and they have to use the private dentists for advance treatment options.

P6, a 30 years old I-Taukei, Male stated:

*“Some of the services are not available here and we have to go to the private clinic which is much more expensive. What’s the use of having this clinic than when it can’t provide the care needed.”*

Similarly, P23, a 55 years old Indian, Male said:

*“They said because of covid they only do pull out of tooth and no filling. So I asked the dentist than what about my tooth which needs filling? He said go to the private dentist, but I told him it would be expensive to which he responded it’s not his problem. Covid or no covid the treatment options at this clinic is very limited and the staff pathetic as usual.”*

Similar sentiments were shared by another participant who stated that treatment would depend on the availability of materials and with basic care:

P19, a 27 years old Indian, Male said:

*“The treatments options here are limited only basic care is provided, that too will depend on the availability of materials. Sometimes it’s a waste of time coming here better go to private clinics with wider options for treatment.”*

Another participant mentioned that some procedures are not available at the dental clinic and they can’t afford private dental treatment thus end up losing their tooth.

P16, a 33 years old Indian, Male mentioned:

*“Today I came to get my tooth pulled out because 5 months ago the dentist here told me to go to the private clinic for a root canal or something she wrote on a paper. I went to the private dentist and was quoted a price of about $900 for the procedure which I could not afford and left my tooth like that until today I had it removed because of the pain. If only that treatment option was available at this clinic my tooth could have been saved.”*

The issue is not of having qualified dentists but less treatment options given by the government to the people was also highlighted.

P9, a 25 years old I-Taukei, Female stated:

*“What’s the use of having so many qualified dentists when the clinic only provides some treatments only? The option is very less, government should look into this as we have qualified people who can give top class treatment with the right resources. The dentists here only know how to pull out teeth, not save them.”*

## Discussion

In the findings of this research the participants identified waiting time before treatment, cost of services, accessibility of services, privacy and confidentiality and range of treatment options as those factors which affect them in terms of the services provided at the CWMH dental clinic.

Waiting time before receiving treatment is an issue which was identified by the majority of the participants in this study. Some of the participants claimed waiting for almost 3 h before being seen by a dentist. This was also mentioned Akbar et al. [16] whereby waiting time on health treatment has been reported as one of the factors that increase the level of frustration in patients and is regarded as an obstacle in one's activities, and this was also seen in the findings of this study whereby some participants had to go to work and planned to visit the clinic early morning so that they could be seen and return to work afterwards, but unfortunately ended up waiting for two hours and mentioned that their salary would be deducted because of the waiting time.

The length of time patients spend in a waiting room has been associated with having negative effect on patient’s perception of the service [17]. In a quasi-experimental study, conducted by Inglehart et al. [17] it was found that letting patients wait for their appointments and not being on time affects their satisfaction negatively. The authors also concluded, that longer waiting time not only affects the patient’s response, but also lowers the satisfaction of the provider, therefore longer waiting times affect the dynamics of the patient provider relationship.

Furthermore, participants also suggested that children needed to be seen first as a 5 year old was made to wait for two hours before being seen by the dentist, while the parent kept pleading to the reception staff that his child’s issue was an emergency. The findings of a study conducted by Fux-Noy, et al. [18] explained that the level of dental anxiety increased in children who were waiting for emergency treatment in comparison to those scheduled for dental treatment who knew that they will be seen by a dentist. A study also found that children’s dental anxiety can be reduced by preventing emergency treatments, scheduling routine dental visits and decreasing waiting time [19].

In terms of the cost of treatment most people do not seek dental care due to the high cost associated with it. In a study conducted by Wallace and MacEntee [20] involving low-income earners, dentists and social service providers in Canada, it was found that low income participants along with dentists and other healthcare providers identified the cost of dentistry and the inadequacy or inaccessibility of public insurance schemes as major impediments to dental services for low income people. Some participants also argued on the fact that the government medical services were provided free of charge and questioned why the dental services was not following a similar approach as the burden of cost was too much for them to bare as they did not earn much but had kids who needed dental care and had to choose who goes to the dentist when they had money to spare for treatment. Amin and Harrison [21] highlighted that despite the reduced cost in public sector, some individuals still find the cost of dental treatment as a barrier to accessing dental care. Some parents expressed that they do not take their children to the dentist as they find it expensive.

Additionally, some participants stated that they would still prefer coming to the CWMH dental clinic since it was a public facility which is cheaper than the private dental clinics who charge exorbitant rates. In a related study conducted by Kadaluru, Kempraj and Muddaiah [22] involving 246 adults aged 18–55 attending community outreach in Bangalore, it was found that 22 percent of the respondent's stated that high cost of oral health care was one of the major barriers for seeking oral health care. In this matter, public hospitals provide dental services at a lower cost compared to private clinics. As such, many people choose public hospitals over private clinics for their dental needs.

Furthermore, this research also established that a few participants with dental issues would rather wait for their tooth to get worse and then turn up at the clinic to get an extraction done which they claim is far cheaper than getting a dental amalgam procedure to save the tooth. This was also mentioned by Glazman [23] whereby some patients who are unable to afford treatment options, deliberately reach a point where their oral health is so compromised that instead of addressing the issue and taking preventive steps, they cause their teeth to be extracted or lost.

In regard to the accessibility of services, accessibility in dental care refers to how easily patients can utilize the dental services provided. Research indicates that access to general dental care is incredibly difficult for individuals with special needs [24]. According to a study done by Marshman et al. [25] a postal survey of 10,864 adults in the United Kingdom found that the perceived difficulty of accessing a dentist was a predictor of oral health outcomes and indirectly affected the use of dental services by perceived need.

The issue of dental chairs not being disabled and elderly friendly was also highlighted whereby participants described that the chairs reclines were not working and some elderly patients claimed that they had to jump onto the chairs because it was high and could not come down, because of that they felt embarrassed and did not want to visit the dental clinic.

According to Wallace and MacEntee [20] cognitive and physical disabilities, compounded by substance use and homelessness, can be serious impediments to accessing treatment in the traditional dental practice. In addition, there are also reports that dentists find difficulties managing patients in wheelchairs or long-term care facilities, or who need sedation. Apart from issues in logistics and accessibility, some studies show that the fear of dental treatment and associated anxiety was identified by many low – income participants as reasons for avoiding dentists, even when public health dental benefits were available.

Similarly, a study conducted by Leal Rocha et al. [26] in Brazil found that the lack of accessibility of health units and their surroundings does not promote the treatment of people with disabilities. Cultural, organizational, architectural, geographical, and communication barriers constrain the demand for and use of oral dental care services by this social segment.

For Privacy and confidentiality, dentists must ensure that they perform their duties such that privacy and confidentiality is upheld. When patients attend a dental practice or clinic, they expect that their data or what they regard as private information will be handled with care by those who might get to know some (or all) of that information [27]. Contrary to this, the findings of this study revealed that at CWMH dental clinic a participant identified an issue of breach of their privacy and confidentiality whereby the dentist who was a relative of the patient and assumed by the patient to have checked their folder and later approached the patient saying he could have done the tooth extraction, which caused embarrassment for the patient. A study in Brazil found that while most dentists (91.43%) believed that the auxiliary cabinet workers were equipped to value patient data confidentiality, 44.29% of those surveyed behave otherwise, discussing clinical cases with individuals outside the dental cabinet [28].

Furthermore, this study found that some other patients identified issues with dentists not closing the door of the dental cubical while attending to the patients at CWMH dental clinic. A patient was embarrassed because another dentist walked into the room where this patient was receiving treatment and started chatting with the dentist giving treatment to this patient while the patient’s mouth was open. The patient did not appreciate this as it affected his/her privacy. According to Brennan [27] confidentiality is not an optional extra in dental practice, but essential to good patient care and treatment thus patients need to be ensured with privacy and confidentiality any medical or dental facility.

In terms of the range of treatment options, it is important that patients are given all options for their oral health needs; this can be achieved by spending enough time with the patient and communicating. According to Aldosari et al. [29] a two-way dialogue that makes patients feel understood and part of the decision-making process can be opened up by allowing patients ample time to share their concerns. Not only can it contribute to greater satisfaction, but it will also help dental staff fulfill the needs and wishes of patients.

The participants of this study identified that they did not have many options on the range of treatment provided at the dental clinic as only basic dental treatment was provided which would depend on the availability of materials and for advance treatment options the patients are referred to the private dental clinics which is much more expensive.

A study done by (Sbaraini et al., 2012) [30], concerning 16 adult patients in Australia, found that even when patients were uncertain about the value of a recommended treatment, a perception that their dentist cared about their problems persuaded them towards compliance. This suggests that even the most “uncooperative” patient may have the potential to be more cooperative in the context of such a relationship. Furthermore, most patients’-based complaints arise because of an imperfect relationship, further accentuating the importance of communication between professionals and their patients [31].

### Study limitations

Due to Covid-19 restrictions it was difficult recruiting patients to participate in the interviews.

## Conclusion

This study shows an overall dissatisfaction with regards to services delivery among patients who are the end users of the services provided by a government facility like CWMH dental clinic. The patients have an expectation to get the best treatment at a reasonable cost, but contrary to this expectation numerous issues in regards to services have been highlighted negatively by the participants of this study. The Ministry of Health and Medical Services (MoHMS) needs to look into the genuine concerns that have been raised by patients in order to create improvements in services delivery and create an array of satisfaction for its patients especially in area of waiting time before treatment, cost of treatment, accessibility of services, privacy and confidentiality and range of treatment options available for the patients. One of the best approaches would be to provide training to staff in the area of customer service. This would need to have a multi-pronged approach with inputs from stakeholders, MoHMS along with staff and managers of the dental clinic to facilitate these changes.

## Data Availability

The data that supports the findings of this study are available on request from the corresponding author.
